# The Impact of Food-Shaping Techniques on Nutrition, Mealtime Experiences, and Quality of Life for Older Adults in Aged Care Settings: A Systematic Review

**DOI:** 10.1007/s13668-023-00493-w

**Published:** 2023-09-04

**Authors:** Lily Chen, Bronwyn Hemsley, Deborah Debono

**Affiliations:** University of Technology Sydney, 15 Broadway, Ultimo, NSW, 2007 Sydney Australia

**Keywords:** Food shaping, Nutrition, Older people, Swallowing difficulty, Systematic review, Dysphagia

## Abstract

**Objective:**

Food-shaping methods, particularly for texture-modified foods, present numerous opportunities to improve the visual appeal of food and potentially the nutrition of older people with dysphagia (swallowing difficulty). This review appraises and synthesizes the evidence on the use of food-shaping methods to enhance nutrition in older adults with swallowing difficulties.

**Methods:**

In August 2022, five electronic databases (PubMed, CINAHL, Scopus, Cochrane Library, and Web of Science) were searched for original research related to the intersection of nutrition, food shaping, and older adults with swallowing difficulties. Characteristics of included studies were described and data from results and findings extracted for analysis across studies.

**Results:**

Eighteen studies met the inclusion criteria and encompassed a variety of settings, including aged care settings (*n* = 15) and food science laboratories (*n* = 3). Qualitative and quantitative findings demonstrated a variety of impacts of food shaping for the older adult with swallowing difficulty, including enhanced nutrition and wellbeing. The content themes identified across studies were: links between food shaping and nutrition, shaping food for individual choice, food shaping for enjoyment, a combination of supporting factors, collaborative inter-disciplinary teams and roles, and implementation aligns with local contexts.

**Conclusion:**

With recent growth in research on food shaping, the body of studies reviewed identified an influence of food-shaping methods on the nutritional status of older adults with swallowing difficulties. Given the identified potential benefit of food shaping and attention to the visual appeal of food for older people, further research examining links between food shaping and nutrition are warranted.

## Introduction

Food shaping has the potential to improve the appeal of food for older adults with swallowing difficulties [1], and such enhancement can increase its consumption [2]. Encouraging the consumption of food is particularly important in residential aged care settings, where it has been estimated that up to 50% of residents are malnourished [3, 4]. Food-shaping methods are an important element of food design and have recently been reviewed for their impact on mealtime-related quality of life [5]. Such food shaping methods include the use of spoons to create rounded or oval shapes, ice-cream scoops, food molds, piping bags, and 3D food printing [6]. Food-shaping methods have been used to create attractive food shapes, and the use of these techniques and technologies is expected to improve mealtime-related quality of life and safety for people with swallowing disability [7]. 3D food printing, in particular, is an emergent food-processing technology proposed to improve the food design of texture-modified food by shortening the traditional trial-and-error stage of the process and automating production of the shaped food [6]. As yet untested in clinical trials with older people or people with swallowing difficulty, it is suggested that 3D food printing could improve nutrition in older people who require texture-modified foods by (a) improving the quality of puree food textures in the production of puree (i.e., by increased standardization) [1], (b) improving the visual appeal of puree foods, and hence encouraging people to eat more of their meals [8], (c) increasing individual choice and control through customization in the design of food items [5], (d) increasing safety by making it easier to produce the correct texture and motivating adherence to texture-modified [9], and (e) enhancing the nutritional content of the printed food [10]. However, there is little empirical research to date on these potential benefits. Furthermore, it is important to identify any food-shaping methods at all that might improve nutrition in residents of aged care facilities who require texture-modified foods. Such knowledge could impact the research and development of new food design technologies at the intersection of advancements in food processing and shaping technologies for people on texture-modified diets (e.g., pureed or minced and moist foods). As such, this is the first systematic review that we know of looking at the impact of any food-shaping method on nutrition in older people, particularly those with swallowing difficulty, with the aim of identifying any potential links between food shape and nutrition to inform both clinical recommendations for this population and future research in this domain for people with swallowing difficulty.

### Older Adults with Swallowing Difficulties

Changes in swallowing associated with aging can affect how the older adult eats food. Age-related changes affecting swallowing function include a more prolonged oral phase, reduced tongue pressure, and delayed triggering of the swallow reflex [11]. If the older adult also has dysphagia, or swallowing difficulty, management of that person’s respiratory and general health and nutrition may become more complex if there is also reduced access to food and nutrients. Dysphagia is associated with a wide range of health conditions which are more prevalent in older age groups (e.g., stroke, Parkinson’s disease, and dementia) [12] and can affect the person’s nutrition and hydration [13]. It is estimated that as many as 58.69% of older people living in aged care facilities have dysphagia [12]. Since malnutrition is accompanied by a loss of muscle mass and function, which also affects masticatory and swallowing muscles, dysphagia can therefore have impacts that further reduce swallowing function over time [11]. Some individuals, even before they know they have dysphagia, adapt to swallowing difficulties by eating smaller amounts, eating more slowly, or avoiding food and liquids that are difficult to swallow [14]. By the time they seek help from a health professional such as a speech-language pathologist or dietitian, their swallowing difficulty might have worsened and nutritional impacts become evident [14].

Older people living in aged care facilities or independently in the community have shown differing degrees of tolerance to texture and taste [15]. When asked to judge the visual appeal of pureed food consistencies, aged care residents and nursing home staff consider puree to be unappealing due to its resemblance to baby food [9, 16] or “slop” [17, p. 88]. Indeed, such pre-conceived notions of the pureed food can undermine the eating experience and cause disappointment [18]. Tailoring food textures, flavors, colors, shape, and presentation to the person’s known anatomical and physiological changes in chewing and swallowing provides novel opportunities in terms of mealtime experiences [19]. Food-shaping methods may serve to directly influence the ingredient composition of the foods and enable the inclusion of custom amounts of nutrients [20]. Through food shaping, older adults with swallowing difficulties may have an opportunity to enjoy foods that nourish them while also being safe to swallow and reducing fears associated with swallowing difficulty such as choking on food [21••].

### Benefits of Food Shaping Interventions

The potential for food-shaping interventions to improve nutrient status could also yield health benefits for the older adult. Shaped foods have been shown to increase the intake of both macronutrients (e.g. proteins and lipids) and micronutrients (e.g. potassium, magnesium, and zinc) in older adults between 65 and 90 years who had been at the facility for 3 months, had either involuntary weight loss greater than 7.5% of usual body weight during the past 3 months, or a BMI of less than 24 [22]. The need for nutritious food is greater for older adults, and those on texture-modified diets have been shown to have a higher level of energy- and protein-deficiency in comparison to those receiving the usual, standard texture meals [23]. The use of nutrition enrichment (e.g. shaped pureed meat and vegetables with the addition of protein drinks or cream) can be a viable solution to personalize food to individual preferences [23].

#### Food Molds

Food molds enable the presentation of pureed food in a visually appealing and familiar ways (i.e., in a shape that resembles the food’s main ingredients such as vegetables, chicken, beef, or fruit); and older adults have been shown to have higher satisfaction with the appearance of molded compared to unmolded pureed foods [24]. Although the acceptance of pureed food shaped with a food mold has been shown to have no impact on first choice and food consumption [25], some studies point to positive results in older adults with swallowing difficulties having a higher acceptance of molded food in comparison with pureed food [9] and enhanced food intake [22, 26, 27•]. Several other factors should be taken into consideration in relation to acceptance of shaped pureed foods (e.g., individual choice and sensory interactions in the mouth) [28, 29]. With staff having training and experience in using food molds, this practice becomes more effective [10] and further improves acceptability.

#### 3D Food Printing

3D food printing has potential application in various sectors, including aged care, in developing the printing of a wider range of natural and nutritious foods for people with swallowing disability [6]. Although there are challenges associated with 3D food printing, such as the intricacies of optimizing the 3D food printing process [8], the use of non-thermal technologies (e.g., high pressure cooking and thermal processing) to prepare foods for 3D printing has some benefits. Such technologies have been shown to maintain bioactive compounds and manipulate rheological properties as a desirable starting material [30–32]. A prior systematic review of the literature relating to 3D printed foods for people with swallowing difficulty [1] identified 16 peer-reviewed papers highlighting the affordances of 3D food printing for improving the visual appeal of pureed foods. However, the vast majority were narrative review papers (*n* = 13) relying on anecdotal reports, promotional materials, and specification documents from 3D food printing companies; with little primary empirical, critical, or original research that included end users or consumers of the food. To be feasible or compete with other food-shaping methods already available at relatively low cost, 3D food printing would need to provide the correct puree texture to meet the International Dysphagia Diet Standardization Initiative (IDDSI) standards [33] and be more appealing and acceptable than other food-shaping techniques [1].

### Aims

In order to identify prior and current areas of strength and knowledge to influence further research on the impact of food shaping on nutrition for older people in residential care settings, it is important to better understand the nutritional and quality of life benefits of food-shaping interventions in aged care settings. Therefore, the aims of this review are to (a) understand the impact of food shaping on nutrition, mealtime enjoyment, and participation in food provision in aged care settings; and (b) explore the intersection of food shaping, nutrition, and older adults with swallowing difficulties. This review is expected to contribute new knowledge on how prior research could (a) drive further innovation in the design of food shaping methods, (b) inform improvements to mealtime participation and quality of life for people living in aged care settings, and (c) inform future developments in the design of texture-modified foods for older people with swallowing difficulty.

## Method

The protocol of this review was published on November 9, 2021 [34]. In August 2022, a systematic search of five scientific databases (PubMed, CINAHL, Scopus, Cochrane Library, and Web of Science) was conducted using search terms related to nutrition and food shaping in various combinations and permutations (Appendix). In this study, an aged care setting was defined as a long-term residential care setting to accommodate older adults (i.e., greater than 65 years of age). Based on the inclusion criteria, only peer-reviewed, original research full papers published in English in the past 20 years were included in the analysis. The full search strategy is available from the first author.

**Fig. 1:**
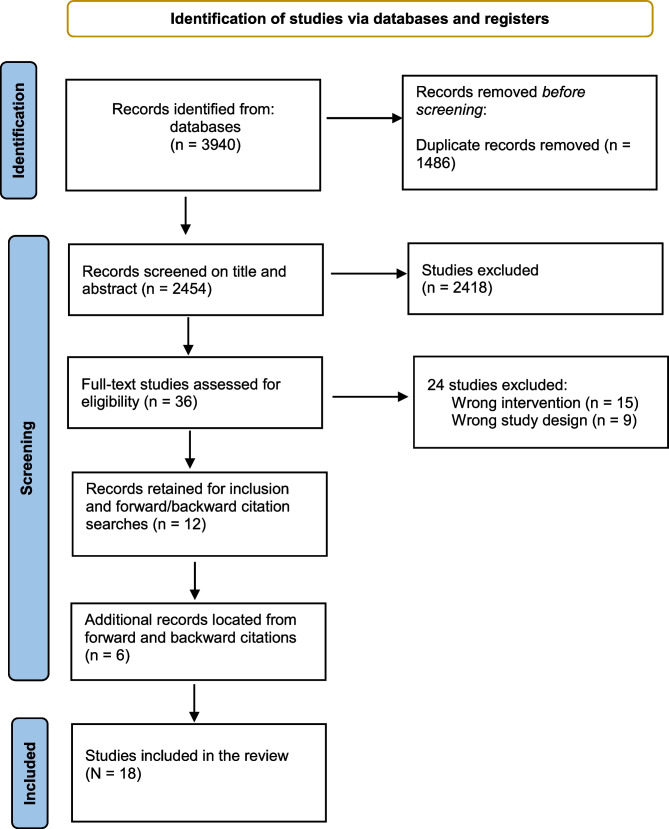
Flowchart for literature identification, review, and exclusion adapted from the PRISMA diagram [35]

Papers identified in the database searches (*n* = 3940) were uploaded into Covidence, an online systematic review software tool, and the duplicates removed (*n* = 1486). The remaining papers (*n* = 2454) were initially screened by title and abstract against the selection criteria by two authors (initials de-identified), and the full texts were retrieved for full review. At all stages, any discrepancies were resolved by the third author. This procedure located 12 studies meeting the inclusion criteria, which were subjected to a forward and backwards citation search to locate any additional relevant studies meeting the inclusion criteria. The forwards and backwards citation search involved the identification, assessment, and inclusion of relevant studies from a hand search of (a) the reference list of each included study, and (b) studies subsequently citing each included study. This resulted in a further six studies being located, and in total, 18 studies were ultimately included in the review. The PRISMA flowchart of search results and included studies is presented in Fig. 1.

## Food Shaping, Nutrition and the Older Adult with Swallowing Difficulty: A Review

### Data Extraction

For each study, the bibliographic information, aims, methods, findings, and outcomes of studies were extracted into an Excel file by the first author and checked by the second author. Data extracted included (a) the study characteristics of author, year, title, aim, methodology, role of nutrition, and type of food-shaping method; (b) method of data collection, data collected, method of data analysis, intervention, findings, outcomes, any content themes reported in the study; and (c) the authors’ reported limitations and directions for future research.

### Method of Analysis

The findings, outcomes, and any content themes reported were analyzed across studies using the integrative review method. The integrative review method was used to combine diverse data through a systematic and rigorous approach consisting of data reduction, data display, data comparison, conclusion drawing, and verification [36]. Heterogeneity in study designs prevented the comparison of findings using statistical methods. Consultation among all authors occurred throughout the process of analysis as studies were read and coded iteratively, individually, and as a group. The first author, an experienced dietitian, used reflexive thematic analysis to code the data based on its content, first with open coding, developing, and in the later stages, applying the emergent analytical framework of content categories and themes and interpreting the data to explore relationships [37]. Reflexive thematic analysis seeks to draw descriptive and explanatory conclusions around themes through identifying commonalities and differences in qualitative data and focusing on relationships between different parts of the data [37]. Thus, the five qualitative studies were analyzed as a group using reflexive thematic analysis and described according to the measures used in the studies and any categorical data. The eight mixed methods studies were analyzed both descriptively and qualitatively. To integrate the analysis across diverse methodologies, extracted data and the content codes applied to each study were arranged into categories of meaning, facilitating the identification of patterns, themes, variations, and relationships [36]. Results are reported with attention to these patterns, and excerpts and quotes are provided to illustrate and support the authors’ interpretations, for increased plausibility and confirmability in the findings.

## Results

### Characteristics and Quality of the Included Studies

The characteristics and appraised quality of the studies are presented in Table 1.

**Table 1:** Characteristics of the included studies

Ref #	First author, (year)	Duration	Location	Participants	Study design	Study methods	Setting	Food-shaping type	Future directions	QATSSD score
[42]	Awwad (2019)	N/A	Canada	39 participants (10 dietitians, 19 dietary workers, 8 residents and 2 family, members)	Qualitative	Interview, discussion group	Aged care	3D food printing	Determine whether 3D food printing is a workable, acceptable, and effective solution	36/42 = 86%
[40•]	Burger (2019)	N/A	Germany	590 nursing homes	Mixed methods	Survey	Aged care	Food shaping (general)	Quality of textured-modified food for residents	23/48 = 48%
[39]	Dahl (2017)	N/A	US	97 aged care facilities	Mixed methods	Survey	Aged care	Food mold	Examine the preferences of long-term care residents with dysphagia for molded pureed foods	23/48 = 48%
[22]	Germain (2006)	12 weeks	Canada	93 aged care residents between 65 and 90 years of age	Quantitative	Observations	Aged care	Food mold	Nutritious and appealing foods for older adults with dysphagia	21/42 = 50%
[24]	Higashiguichi (2013)	7 days	Japan	57 aged care residents whose energy requirements were evaluated at 1200–1500 kcal	Mixed methods	Observations, survey	Aged care	Food shaping (general)	Not specified	25/48 = 52%
[29]	Houjaji (2009)	N/A	Canada	N/A	Mixed methods	Measurements	Laboratory	Food mold	Texture measurement be done at ‘in-mouth’ environment to study the therapeutic efficiency of modified texture foods	33/48 = 69%
[41]	Ilhamto (2014)	N/A	Canada	25 aged care facilities	Qualitative	Interviews	Aged care	Food mold	Modifications to recipes and products to demonstrate the tailoring of pureed food is a preferred practice	30/42 = 71%
[43]	Keller (2014)	10 months	Canada	80 allied health professionals	Qualitative	Focus groups	Aged care	Food mold	Focus on staff education and how to promote flexibility to meet individualized needs	31/42 = 74%
[44]	Kimura (2019)	N/A	Japan	Experiment 1 (21 aged care residents with dementia, aged 73–93); Experiment 2 (14 aged care residents with dementia, aged 73–93 years)	Quantitative	Survey	Aged care	Addition of sauce	Further studies that control for familiarity with or eating habit of wiping up the sauce with food	26/42 = 62%
[46]	Lepore (2014)	N/A	US	70 aged care residents	Mixed methods	Survey	Laboratory	Food mold, scoops	The acceptability of pureed foods and the impact of shaping pureed foods on intake of individuals with dysphagia	17/42 = 40%
[28]	Milte (2017)	N/A	Australia	13 aged care residents, 6 family members	Qualitative	Interview	Aged care, community	Food mold	Strategies to support implementation of interventions to improve the mealtime experience for residents	33/42 = 79%
[38]	Milte (2018)	4 months	Australia	204 aged care facilities	Quantitative	Survey	Aged care	Food mold	Strategies to support implementation of interventions to improve the mealtime experience for residents	23/42 = 55%
[47]	Okkels (2018)	N/A	Denmark	30 aged care residents with dysphagia; aged older than 70 years	Quantitative	Observations and measurements	Aged care	Layering	Repeat the study with a larger group of participants and for a longer period to ensure validity of results	24/42 = 57%
[26]	Ott (2019)	Two phases each lasting 6 weeks	Germany	16 aged care residents with chewing/swallowing problems; who received texture-modified food regularly	Mixed methods	Interview, nutrition assessment	Aged care	Food mold	Further research to confirm positive effects in large samples	19/48 = 40%
[25]	Pouyet (2014)	4 weeks	France	114 aged care residents and 19 aged care professionals	Mixed methods	Interviews, questionnaires	Aged care	Food mold	Development of new foods for aged care	38/48 = 79%
[27•]	Pu (2021)	Two phases each lasting 3 weeks	Australia	28 aged care residents who showed signs and symptoms of dysphagia and ate texture-modified diets with one puree texture for at least 6 months prior	Mixed methods	Training workshop, observations	Aged care	Food mold	Larger sample size and breakdown of textural properties of modified food	19/48 = 40%
[21••]	Smith (2022)	3 months	Australia	15 allied health professionals (12 speech-language pathologists, 3 occupational therapists)	Qualitative	Focus group	Aged care (online)	3D food printing	Examine how dietitians and nutritionists, people with dysphagia and their supporters engaged with a 3D food printer and their views on the acceptability or use of these devices	30/42 = 71%
[45]	Van Eck (2019)	4 weeks	Netherlands	17 adults aged 20 to 34 years old	Quantitative	Observations and measurements	Laboratory	Pre-cutting	To develop a full picture of vegetable intake and subsequent nutrient absorption	25/42 = 60%

Years of publication ranged from 2006 to 2022, with 9 (50%) published in the last 5 years. The five quantitative, five qualitative, and eight mix-method studies related to settings including aged care settings (*n* = 15) and food science laboratories (*n* = 3). The duration of the studies ranged from 7 days to 10 months and the sample sizes across studies ranged in both number and type (e.g., aged care residents, healthcare professionals, and aged care settings). For residents in aged care settings, the researchers had reported on age ranges, medical conditions (e.g., dementia), energy requirements, and length of time receiving texture-modified foods.

### Quality Appraisal, Strengths, and Limitations of the Included Studies

The quality of the included studies was assessed by the quality assessment tool for studies with diverse design (QATSDD) [48]. This tool is considered valuable in its ability to draw quality assessment across a diversity of sources of evidence to gauge the integrity and reliability holistically [48]. A total of 16 criteria is included, where 14 of the criteria apply to qualitative studies, 14 apply to quantitative studies, and all 16 apply to mixed methods [48]. Each criterion is scored ranging between 0 and 3 which allots the maximum possible score of 42 for qualitative or quantitative studies and 48 for mixed-method studies. For each included study, criteria scores were added and divided by the maximum possible score (%QATSDD total score) to report the overall quality score. The first author applied QATSDD to the included studies which were reviewed by the second and third authors.

The strengths of the qualitative studies included the examination of respective research questions to be examined in detail and the use of multiple methods to verify and expand on findings [21••, 25, 28, 39, 41, 43] and to develop findings from one phase to another [e.g., 29, 40•]. Mixed methods studies where the diverse data points captured were better able to capture the role of food shaping to improve nutrition in aged care, such as both mealtime observations [27•], observations with measurements to find enhanced energy, and protein intake with enriched and reshaped texture-modified diets [26], or with questionnaires [24] and feedback from nursing personnel [26] or older adults with dysphagia [47], lab tests of foods combined with acceptability ratings [46], or surveys on meal choices [38].

The limitations included the lack of detailed information regarding the recruitment or discussion of self-selection bias that may impact the findings [25, 28, 39, 43], or not specifying the numbers of participants in sub-groups [42], or the use of homogenous groups which may have restricted the dynamics of the group discussion in capturing a wide range of perspectives [e.g., 21••, 41]. A further limitation related to the lack of detailed information regarding the accuracy of measurements taken in the studies (e.g., use of a scale lift of chair, knee height measurements for height, nutrient analysis program to measure dietary intake, differential weight measures to measure items served on a tray before and after each meal [22], or video analysis [45]). There was a lack of information regarding the maintenance and calibration of measurement tools to ensure validity. Limitations are the constructs being measured are inherently difficult to capture accurately such as people’s values and beliefs (e.g., subjective ratings being affected by participants’ difficulties in understanding due to reduced cognitive function [27•]). Studies using surveys without interviews may have restricted the respondents’ elaboration of the nuances of food shaping on taste, experience, and enjoyment [24, 38, 40•, 47].

### Aims of the Included Studies

While all studies were related to the process of food shaping and its impacts on nutrition, mealtime enjoyment, and participation in food provision in aged care settings, the aims of the included studies varied widely; from the development or testing of food shapes in the laboratory to implementing and evaluating food-shaping techniques in residential care settings. The foci of the studies were diverse (i.e., from the bench to the table), encompassing (a) the creation of food-shaping being understood through the identification of textural parameters, which ultimately influence shaping properties [29], and (b) understanding how food shaping for older adults in residential care might be improved, through the knowledge of how food and dining is currently provided in aged care settings [38, 39] and the implementation of texture-modified diets [40•]. Understanding the challenges and strategies involved in the provision of texture-modified food can also help to identify and inform preferred practices [41]. The acceptance of food shaping can also be better understood by exploring how long-term residents [27•, 28, 42] and healthcare providers [21••, 43] perceive food shaping.

Adjusting even one factor (i.e., the shape of the food) may potentially alter its acceptability to older people or their service providers. The consumer’s perceptions of food shaping may be influenced by several other factors, such as the addition of sauce [44], condiments [45] and serving style [46]. The influence of appearance on diet [24] and shape on attractiveness on food [25] influences the acceptability of the food to older adults based on sensory properties [47]. Some authors identified that the knowledge of the effects of innovative texture-modified food [26] improves the nutrient intake of aged care residents [22].

The largest group of studies (*n* = 8) focused on the intersection of food shaping, nutrition, and older adults with swallowing difficulties from multiple perspectives; notably allied health professionals [21••, 43] and long-term care residents [25, 27•, 28, 42, 44, 47]. A smaller group of studies (*n* = 4) focused on how food shaping may impact the older adult’s clinical nutrition status, including dietary intake [22, 26], changes in nutrient intake (e.g., energy, protein, and lipids) based on the consumption rate [24], and the current practices of preparation and purchase of modified-texture foods to improve food intake and nutrition [39]. In total, only three studies examined food service systems including the best practices of providing texture-modified food [40•], the current food and dining characteristics in Australia [38], as well as the challenges involved in facility-produced pureed food [41]. Finally, three studies examined food shaping in the food science laboratory setting with a focus on sensory properties of the food sample assessed. More specifically, evaluating the impact of serving style (e.g., scooped versus molded pureed meats and vegetables) on the identification and acceptability of pureed foods [46], the effect of shape on eating behavior towards carrots [45], and the quantification of the textural parameters of therapeutic foods in the form of cakes [29].

### Analysis of the Results of the Included Studies

The main results informing in the analysis are presented in Table 2, and an overview of how included studies contributed to the content themes and categories within each theme are presented in Table 3. Each of the content themes is explained in this section, with data, excerpts, and quotes from the included studies provided to increase plausibility and confirmability of the analysis across studies.

**Table 2:** Results of the included studies

Ref #	First author (year)	Results
[42]	Awwad (2019)	• Weighing the risks against the benefits of pureed food with residents and families • Using 3D printing to market pureed food more positively to residents, families, and team members • Finding solutions that are workable • Working through logistics: procurement, storage, retherming, and service • Key considerations for creating a quality product • Piloting with staff, residents, and families
[40•]	Burger (2019)	• Re-shaped components considered by 27.9% of respondents— the least common best practice for nursing home residents
[39]	Dahl (2017)	• Pureed foods were served in 96 homes (99%) to a mean of 17 residents on a given day, which represented 14% of total long-term-care residents • 14 (14.6%) of facilities reported purchasing commercial pureed foods • At most facilities (*n* = 83, 86.5%), pureed foods were “always” prepared from scratch, whereas 7.3% (*n* = 7) and 2.1% (*n* = 2) of facilities prepared pureed food from scratch “sometimes” and “never” respectively • In 73.9% (*n* = 70) of the facilities, standardized recipes “often” and 6.2% (*n* = 6) “sometimes” respondents at 10 (10.4%) of the facilities did not answer • The majority (*n* = 82, 85.4%) indicated pureed foods from scratch was most economical • 3 facilities (3.1%) reported purchasing molded purees • In-house molding of pureed foods was carried out in 14 facilities (14.6%) with meats, vegetables, breads being the food commonly molded • 19 respondents provided comments which centered around 3 issues: (i) the expense, time, and difficulty required for molding purees; (ii) the challenge of achieving standardized thicknesses (viscosities) for thickened beverages; and (iii) low-sodium puree foods, in that the regular-texture foods used to make the purees were lower in sodium, so there was no need to specifically prepare low-sodium purees
[22]	Germain (2006)	• An increase in total energy, protein, lipid, total saturated and monounsaturated fats, potassium, magnesium, calcium, phosphorus, zinc, riboflavin, and vitamin D were significant for the treated group • Higher dietary intake appears to come from higher portions of reformed pureed cakes, reformed pureed vegetables, and reformed pureed meats
[24]	Higashiguichi (2013)	• The study diet showed higher levels of mean energy and protein intake than the modified traditional diet • The dietary nutrition intake per weight was higher for the study diet than for the modified traditional diet • The study diet was an efficient diet rich in nutrients in a smaller quantity of food
[29]	Houjaji (2009)	• Despite their relatively large variations in firmness and springiness, these therapeutic cakes appeared to be fit for dysphagic diets which may be attributed to heat-sensitive binding agent • From a sensorial point of view, the developed therapeutic cakes needed to be soft, aerated, and cohesive enough to retain the shape of the sample and not adhesive in order to prevent sticking to the inner parts of the oral cavity, and slowing its movement downward through the throat • Heat-sensitive binding agents melt almost immediately when placed in a warm environment such as the mouth. Interactions with bodily fluids, saliva, help speed their breakdown and melting process
[41]	Ilhamto (2014)	• Difficulty in using standardized recipes • Varied interpretation of government guidelines • Lack of consistency in terminology and texture • Wanting to improve visual appeal
[43]	Keller (2014)	• Rationalizing the use of pureed foods in accepting of a less than desirable product for the safe swallowing and nutritional health • Providing the right texture was paramount in the providers’ priorities with respect to pureed food, quality of life was secondary • Affirming the providers’ self-concept as nurturers, and providers was linked to the quality of the pureed food products they provided to residents/patients • Doing whatever it takes which describes how staff manipulated or worked within the constraints of food production to meet the needs and preferences of their residents/patients • Making improvements to promote the entire sensory experience for residents/patients and enhance food intake
[44]	Kimura (2019)	Experiment 1 • Mean amount of snacks with chocolate sauce consumed was 7.8 pieces (82.8% of the mean total amount) • 90.5% of participants ate more snacks with sauce than without sauce and 81% dipped snacks without sauce in remaining sauce and ate them • 19 participants had greater intake of snacks with sauce than without sauce Experiment 2 • Mean amount of snacks with agave sauce (low visual contrast) was 6.9 pieces (77.9% of mean total amount) • 64.3% of participants ate more snacks with sauce than without sauce, 50% dipped snacks without sauce in remaining sauce and ate them • 9 participants had greater intakes of snacks with sauce than without sauce
[46]	Lepore (2014)	• The mean rating of the participants’ favorite foods was 60 for younger adults and 65 for older adults on the scale from − 100 to + 100 • The overall acceptability of scooped and molded purees approached neutral on Labeled Magnitude Scale (HgLMS) • Correct food identification ranged from 15 to 59% • Correct food plus food group identification ranged from 63 to 96%
[28]	Milte (2017)	• People with cognitive impairment in nursing homes seek to have their individual needs and preferences recognized and heard, expressed frustration as they perceived growing barriers to receiving dietary care which met preferences, and described a deterioration of the amount of control and choice available to the individual with loss of self-feeding ability and dysphagia
[38]	Milte (2018)	• High contrast plates (*n* = 51, 25%) and molds to reform texture-modified meals (*n* = 41, 20.1%) were used by no more than a quarter of facilities
[47]	Okkels (2018)	• Significant correlation between mean flavor preference and fat and energy content which are high importance old adults with dysphagia • The most liked in-between meals were all dessert (cold, frozen, and predominantly sweet)
[26]	Ott (2019)	• Energy intake increased by 204.2 kcal and 18.3 g protein from W6 to W8 after Period 2 enriched/reshaped textured-modified diet
[25]	Pouyet (2014)	• Finger foods with a sauce (vs no sauce) and with two layers (vs one layer) were more frequently chosen first and then consumed • The shape (molded vs squared) of the finger foods had no impact on first choice and food consumption • Interviews revealed three parameters to have a potential impact on food attractiveness because they could address changes related to age and disease: shape, presence of sauce, and visual contrast • According to interviewees, shape is important to optimize food grasping (adaption to physical disorders) and the visual appeal of finger foods
[27•]	Pu (2021)	• 7.6% increase in mean oral intake amount per meal for modified purees compared to traditional purees
[21••]	Smith (2022)	• 4 main content themes identified: (i) Visual appeal: the impact of the visual appearance of 3D-printed food; (ii) Costs: the financial and time costs involved in 3D food printing; (iii) Practicality: the practicality of people with dysphagia or their supporters using the 3D food printer; and (iv) Potential: the potential for 3D printing to be a "technology for good" in improving mealtime-related quality of life
[45]	Van Eck (2019)	• Cutting carrots into julienne shape increases rather than reduces mastication effort • Addition of mayonnaise increased carrot particle sizes at the moment of swallowing, thus, a decrease in carotene bioaccessibility can be expected • With the addition of lipids, carotenoid accessibility is increased

**Table 3:** Table of the included studies’ findings contributing to the content themes

Ref #	First author, year	Contribution to theme	Theme
[42]	Awwad (2019)	The fortification of shaped food with vitamins and minerals may improve nutritional quality of food, particularly in studies relating to 3D food printing	Links between food shaping and nutrition
		The possibility of increasing access to nutritionally fortified pureed foods was a major factor dietitians identified to drive the uptake and use of 3D food printing	Links between food shaping and nutrition
		Reportedly, the achievement of the highest standards will involve the advancement food shaping, which relies on the support from all levels of healthcare providers who are knowledgeable of the importance of food shaping	Collaborative inter-disciplinary teams and roles
		Dietitians are well-placed to merge food shaping and nutrition interventions together; and see the potential value of 3D food printing as a creative and innovative solution to market pureed food pending continued testing and benchmarking to ensure effectiveness	Collaborative inter-disciplinary teams and roles
		The successful implementation of food shaping methods is helped by close alignment with the setting’s local current practices	Implementation aligns with local contexts
[40•]	Burger (2019)	Facilities implementing a greater number of “best practices” in terms of food provision and a greater variety of textured-modified food types (e.g., pureed, minced, soft, and bite size) are more likely to have greater dietetic and nutrition support	Collaborative inter-disciplinary teams and roles
		Dietitians are well-placed to merge food shaping and nutrition interventions together; and see the potential value of 3D food printing as a creative and innovative solution to market pureed food pending continued testing and benchmarking to ensure effectiveness	Collaborative inter-disciplinary teams and roles
[39]	Dahl (2017)	The shape of the food is only one factor, and if pureed foods are not acceptable in terms of flavor and texture, older people will be at risk of poor food intake, malnutrition, and all associated complications	Links between food shaping and nutrition
		Standardized recipes may not useful for pureed food because properties of foods (e.g., shape, size, and addition of condiments) will likely be different from those on which standard recipes are based	Collaborative inter-disciplinary teams and roles
		The successful implementation of food-shaping methods is helped by close alignment with the setting’s local current practices	Implementation aligns with local contexts
		Although widely available, the use of food molds in residential aged care facilities is uncommon, given failed attempts at molding and concerns about the added expense for molded meals	Implementation aligns with local contexts
[22]	Germain (2016)	Texture-modified purees were shown to improve nutritional status	Links between food shaping and nutrition
[24]	Higashiguichi (2013)	Texture-modified meals could decrease the amounts of nutrients in the food due to the cooking process (e.g., addition of water to soften the food and removal of components that are difficult to eat)	Links between food shaping and nutrition
		As evaluated by the healthcare professionals, changes in the appearance of a meal brought about by modification of the texture (e.g., by mincing or pureeing) has been shown to reduce the joy of eating and the satisfaction level of the older residents in both inpatient hospital and aged care	Enjoyment
		Environmental factors during mealtimes may also positively or negatively influence food intake and nutritional status for older people in residential care	A combination of supporting factors
[29]	Houjaji (2009)	The older adult’s swallowing and oral sensory needs, and need for texture-modified foods, also influences what food shaping methods might be suitable for an individual	Shaping food for individual choice
[41]	Ilhamto (2014)	Positive comments by staff about the food that can enhance food intake which shows the impact of staff behavior	Collaborative inter-disciplinary teams and roles
		Standardized recipes may not useful for pureed food because properties of foods (e.g., shape, size, and addition of condiments) will likely be different from those on which standard recipes are based	Collaborative inter-disciplinary teams and roles
		Reportedly, the achievement of the highest standards will involve the advancement food shaping, which relies on the support from all levels of healthcare providers who are knowledgeable of the importance of food shaping	Collaborative inter-disciplinary teams and roles
		The implementation of food shaping techniques will also affect and be affected by food-service practices, and a number of food service challenges, such as the use of standardized recipes that might or might not be well-suited to creating a pureed texture, varied interpretation of government guidelines, lack of consistency in terminology and texture, and wanting to improve visual appeal	Implementation aligns with local contexts
[43]	Keller (2014)	The fortification of shaped food with vitamins and minerals may improve nutritional quality of food, particularly in studies relating to 3D food printing	Links between food shaping and nutrition
		The older adult’s swallowing and oral sensory needs, and the need for texture-modified foods, also influences what food shaping methods might be suitable for an individual	Shaping food for individual choice
		Staff play a key role in communicating the importance of any necessary food texture modification with residents and family	Collaborative inter-disciplinary teams and roles
		Because of fluctuations of the sensory properties of pureed food (e.g., water and fat separation), a degree of flexibility should be provided for staff members such as cooks to experiment when preparing meals	Collaborative inter-disciplinary teams and roles
		Reportedly, the achievement of the highest standards will involve the advancement food shaping, which relies on support from all levels of healthcare providers who are knowledgeable of the importance of food shaping	Collaborative inter-disciplinary teams and roles
		Establishing the purpose of introducing a new food shaping technique for pureed food is important, with an emphasis not only on safety and nutritional content, but also sensory appeal to the resident; the latter of which has the greatest impact on the residents’ mealtime related quality of life	Implementation aligns with local contexts
		Assessment and review of assessment of the resident’s need for pureed food should also be reviewed to determine when it is no longer required	Implementation aligns with local contexts
[44]	Kimura (2019)	Familiarity with foods is a critical factor in food choice and preference	Shaping food for individual choice
		From the older adult’s perspective, flavor should not differ from what is expected based on its appearance	Enjoyment
		Visual familiarity is key for acceptance of food shaping and preference	A combination of supporting factors
		Some older people are used to eating dishes with sauces, and this intimate familiarity helps to support older adults in this type of food shaping	A combination of supporting factors
		Food accessibility, such as how food is arranged on a single dish, affects food choice among elderly people with dementia	Implementation aligns with local contexts
[46]	Lepore (2014)	Molded purees have been shown to be acceptable to older adult	Links between food shaping and nutrition
[28]	Milte (2017)	An individual’s choice and control over the shape of their food and having access to familiar food shapes is important because food has a physiological effect and an effect on social and quality of life outcomes	Shaping food for individual choice
		An approach to food shaping that lacks flexibility to a person’s individual oral sensory and mastication needs may impact negatively on the person's level of independence, dietary intake, and quality of life	Shaping food for individual choice
		A person-centered approach for the individualization or customization of food shaping is needed and can be achieved by determining what the older adults prefer in terms of food presentation, that is safe to eat	Shaping food for individual choice
		The joy of eating and satisfaction may translate into nutritional benefits if the right food is provided to the resident to enjoy and if individual choice is respected	Enjoyment
		Dietitians are well-placed to merge food shaping and nutrition interventions together; and see the potential value of 3D food printing as a creative and innovative solution to market pureed food pending continued testing and benchmarking to ensure effectiveness	Collaborative inter-disciplinary teams and roles
		Increasing the number of healthcare providers available to manage dysphagia and nutrition in aged care facilities (e.g., speech pathologists, dietitians) could provide more individual assessments of resident capabilities and facility implementation of food shaping that attends to sensory appeal	Implementation aligns with local contexts
[38]	Milte (2018)	While food shaping itself may be beneficial, it is also necessary for people at risk of nutritional compromise receive appropriate nutritional support and advice from a qualified dietitian	Links between food shaping and nutrition
		Dietitians with experience and expertise should be on hand to provide advice to improve the older person’s nutritional intake	Links between food shaping and nutrition
		In contemporary residential aged care settings, as the level of food texture modification increases from soft food to minced and moist or pureed foods, the range of choices offered to the resident eating these foods is reduced	Shaping food for individual choice
[47]	Okkels (2018)	The older adult’s swallowing and oral sensory needs, and need for texture-modified foods, also influences what food shaping methods might be suitable for an individual	Shaping food for individual choice
		In a study of 30 older adults aged 70 years or older with dysphagia, mealtime enjoyment was reportedly related to food presentation stimulating the appetite and enhancing nutritional intake	Enjoyment
		Visual familiarity is key for acceptance of food shaping and preference	A combination of supporting factors
[26]	Ott (2019)	The combination of enrichment, reshaping, and a varied intake all correlated with increases in energy and protein intake	Links between food shaping and nutrition
		Complementary factors need to be used together through the combination of enrichment, reshaping, and a varied intake which correlated with increases in energy and protein intake	A combination of supporting factors
[25]	Pouyet (2014)	Familiarity with foods can influence the foods chosen or selected for consumption	Shaping food for individual choice
		Finger foods with sauce or in two layers, and in particular shapes, promoted food attractiveness and consumption, given its capacity to meet sensory needs and provide cognitive cues to consumption	A combination of supporting factors
[27•]	Pu (2021)	7.6% increase in mean oral intake amount per meal for modified purees compared to traditional purees	Links between food shaping and nutrition
[21••]	Smith (2022)	For novel methods such as 3D food printing, the shaped food should look like the original food product rather than nonfood-like shapes	A combination of supporting factors
		Reportedly, the achievement of the highest standards will involve the advancement food shaping, which relies on support from all levels of healthcare providers who are knowledgeable of the importance of food shaping	Collaborative inter-disciplinary teams and roles
		Dietitians are well-placed to merge food shaping and nutrition interventions together; and see the potential value of 3D food printing as a creative and innovative solution to market pureed food pending continued testing and benchmarking to ensure effectiveness	Collaborative inter-disciplinary teams and roles
		There are further concerns about the usability of the 3D food printer (e.g., time required to prepare or print foods, clean the device, reheat the food, and ensure suitable transportation of the food from production to the table to keep its shape)	Implementation aligns with local contexts
		The limited range of 3D food printers with the capability of printing pureed foods have a relatively high financial cost when compared with the current food-shaping technologies (e.g., food molds and piping bags), and this high cost is a substantial barrier to the uptake	Implementation aligns with local contexts
[45]	Van Eck (2019)	The older adult’s swallowing and oral sensory needs, and need for texture-modified foods, also influences what food shaping methods might be suitable for an individual	Shaping food for individual choice
		Personalizing food shapes can start at the very basic level through everyday essentials such as the knife	Shaping food for individual choice
		The provision of condiments (e.g., cream, sauces) can also influence the visual appeal of a texture-modified meal and, particularly with fat, its nutritional value	A combination of supporting factors
		Standardized recipes may not useful for pureed food because properties of foods (e.g., shape, size, addition of condiments) will likely be different from those on which standard recipes are based	Collaborative inter-disciplinary teams and roles

#### Links Between Food Shaping and Nutrition

Four studies demonstrated food shaping to be feasible for use in a variety of settings [22, 27•, 39, 46]. Molded purees have been shown to be acceptable to older adults [46], with clinical nutrition benefits, as reshaped, texture-modified purees were shown to increase oral intake [27•] and nutritional status [22]. The shape of the food is only one factor, and if pureed foods are not acceptable in terms of flavor and texture, older people will be at risk of poor food intake, malnutrition, and associated complications [39]. Ott et al. [26] demonstrated that the combination of enrichment, reshaping, and a varied intake all correlated with increases in energy and protein intake.

The fortification of shaped food with vitamins and minerals was identified as another option to improve nutritional quality of pureed foods [42], particularly in studies relating to 3D food printing [e.g., 42, 43]. Textured-modified meals could decrease the amounts of nutrients in the food due to the cooking process (e.g., addition of water to soften the food and removal of components that are difficult to eat) [24]. An innovative enzyme-infusion method has shown to adjust the entire meal to a homogenous softness [24]. The possibility of increasing access to nutritionally fortified pureed foods was a major factor dietitians identified to drive the uptake and use of 3D food printing [42]. While food shaping itself may be beneficial, it is also necessary for people at risk of nutritional compromise to receive appropriate nutritional support and advice from a qualified dietitian [38]. Milte et al. [38] highlighted that dietitians with experience and expertise should be available to provide advice to improve the older person’s nutritional intake.

#### Shaping Food for Individual Choice

An individual’s choice and control over the shape of their food and having access to familiar food shapes are important because food has not only a physiological effect but also importantly an effect on social and quality of life outcomes [28]. Familiarity with foods is also a critical factor in food choice and preference [44] and can influence the foods chosen or selected for consumption [25]. In contemporary residential aged care settings, as the level of food texture modification increases from soft food to minced and moist or pureed foods, the range of choices offered to the resident eating these foods is reduced [38]. Moreover, food-shaping tools that are currently readily available (e.g., food molds, ice cream scoops) are under-utilized or not being used effectively in aged care settings [38].

The older adult’s swallowing and oral sensory needs, and need for texture-modified foods, also influence what food shaping methods might be suitable for an individual [29, 43, 45, 47]. The whole process involves oral mastication of food during bolus preparation, transportation through the oral pharyngeal tract, digestion, and absorption of nutrients [29]. An approach to food shaping that lacks flexibility to a person’s individual oral sensory and mastication needs may impact negatively on the person’s level of independence, dietary intake, and quality of life [28]. A person-centered approach for the individualization or customization of food shaping is needed and can be achieved by determining what the older adults prefer in terms of food presentation that is safe to eat [28]. Personalizing food shapes can start at the very basic level through everyday essentials such as the knife (e.g., different cuts of carrot) [45].

#### Enjoyment

Enjoyment of food as an important aspect of meals for older people appeared as a theme across four of the studies [24, 28, 47]. In a study of 30 older adults aged 70 years or older with dysphagia, mealtime enjoyment was reportedly related to food presentation stimulating the appetite and enhancing nutritional intake [47]. From the older adult’s perspective, flavor should not differ from what is expected based on its appearance [44]. However, not all aspects of the visual presentation made a difference to mealtime enjoyment. For example, sprinkling food with green herb, a purple or red berry dust, or arranging food in layers, did not correlate with the enjoyment of a meal [47]. Transparency (i.e., that the food tastes like what it looks like) and familiarity with the foods were highly valued by older people [47].

As evaluated by the healthcare professionals, changes in the appearance of a meal brought about by the modification of the texture (e.g., by mincing or pureeing) has been shown to reduce the joy of eating and the satisfaction level of the older residents in both inpatient hospital and aged care [24]. The joy of eating and satisfaction may translate into nutritional benefits if the right food is provided to the resident to enjoy and if individual choice is respected [28].

#### A Combination of Supporting Factors

The implementation of food-shaping techniques relies upon multiple supporting factors being in place and combined at the level of the individual (e.g., acceptability and preferences), teams (e.g., health professionals and carers), and at the systems level of food service operations. At the individual level, visual familiarity is the key for acceptance of food shaping and preference [44, 47]. Smith et al. [21••] noted that for novel methods such as 3D food printing, the shaped food should look like the original food product rather than nonfood-like shapes. These complementary factors need to be used together [44] through the combination of enrichment, reshaping, and a varied intake which correlated with increases in energy and protein intake [26].

While two of the studies focused on 3D food printing [21••, 42], Pouyet et al. and Van Eck et al. [25, 45] also demonstrate that food shaping does not require complicated technology. Indeed, food shaping and design can take the form of food being cut up [45] or through the simple addition of sauce [25]. Adding sauce to solid food both moistens foods, which may ease chewing and swallowing and aid the passage of food through the digestive system [44]. Sauces can also be used to enhance the flavor, visual color contrasts, and textures of the food on the plate; increasing the resident’s food options for more variety and interest. Some older people are used to eating dishes with sauces, and this intimate familiarity helps to support older adults in this type of food shaping [44]. In addition, presenting food in two layers resulted in residents with dementia in an aged care setting eating more of the food [44]. Also, given its capacity to meet sensory needs and provide cognitive cues to consumption, finger foods with sauce or presented in two layers, and as particular shapes, promoted food attractiveness and consumption [25].

The provision of condiments (e.g., cream, sauces) can also influence the visual appeal of a texture-modified meal and, particularly with fat, its nutritional value [45]. For example, providing a condiment (e.g., mayonnaise) with vegetables shortens mastication time and increases eating rate and vegetable intake [45]. Providing condiments may therefore increase opportunities to facilitate greater food and nutrient intake. Environmental factors during mealtimes may also positively or negatively influence food intake and nutritional status for older people in residential care [24]. For example, the number of people present, food accessibility, eating locations, ambient temperature and lighting, the aroma or smell of food, time of consumption, and ambient sound may all influence intakes [24]. Thus, attention to ensure that environmental, ambient factors are appropriate for the individual is important to increase consumption rate [24].

#### Collaborative Inter-Disciplinary Teams and Roles

The acceptance of food shaping needs the support of many diverse stakeholders, including residents, family members, health professionals, aged care service providers, and the government [41]. The staff plays a key role in communicating the importance of any necessary food texture modification with residents and family [43]. Positive comments by the staff about the food can enhance food intake which shows the impact of staff behavior [41]. Staffs who are supportive are well-positioned to address any difficulties with implementation and identify solutions to promote the increased intake of appealing texture-modified foods [41, 43].

The texture and consistency of pureed food are important for optimal visual appearance. Standardized recipes may not be useful for pureed food because properties of foods (e.g., shape, size, and the addition of condiments) will likely be different from those on which standard recipes are based [39, 41, 45]. Because of fluctuations in the sensory properties of pureed food (e.g., water and fat separation), a degree of flexibility should be provided for staff members such as cooks to experiment when preparing meals [43].

Several studies [21••, 28, 40•, 41–43] provide findings that inform the evidence-base for aged care facilities in relation to food shaping interventions. Facilities implementing a greater number of “best practices” in terms of food provision and a greater variety of textured-modified food types (e.g., pureed, minced, soft, and bite size) are more likely to have greater dietetic and nutrition support [40•]. Reportedly, the achievement of the highest standards will involve the advancement food shaping, which relies on support from all levels of healthcare providers who are knowledgeable of the importance of food shaping [21••, 41–43]. Dietitians are well-placed to merge food shaping and nutrition interventions together and see the potential value of 3D food printing as a creative and innovative solution to market pureed food pending continued testing and benchmarking to ensure effectiveness [21••, 28, 40•, 42].

#### Implementation Aligns with Local Contexts

The successful implementation of food shaping methods is helped by close alignment with the setting’s local current practices [39, 42]. Establishing the purpose of introducing a new food-shaping technique for pureed food is important, with an emphasis not only on safety and nutritional content but also sensory appeal to the resident; the latter of which has the greatest impact on residents’ mealtime-related quality of life [43]. Pureed foods are often recommended out of caution and in response to symptoms of swallowing difficulty, particularly if in the oral phase chewing and forming of the bolus is impaired, to reduce the older person’s risk of choking [28]. Increasing the number of healthcare providers available to manage dysphagia and nutrition in aged care facilities (e.g., speech pathologists and dietitians) could provide more individual assessments of resident capabilities and facility implementation of food shaping that attends to sensory appeal [28]. Assessment and review of the assessment of the resident’s need for pureed food should also be reviewed to determine when it is no longer required [43].

The implementation of food-shaping techniques will also affect and be affected by food service practices and a number of food service challenges, such as the use of standardized recipes that might or might not be well-suited to creating a pureed texture, varied interpretation of government guidelines, lack of consistency in terminology and texture, and wanting to improve visual appeal [41]. If a majority of pureed foods are prepared in-house using standardized puree recipes [39], it is important to assess the efficacy of the practice to minimize challenges and ensure effective food service delivery. Although widely available, the use of food molds in residential aged care facilities is uncommon, given failed attempts at molding and concerns about the added expense for molded meals [39]. The limited range of 3D food printers with the capability of printing pureed foods have a relatively high financial cost when compared with the current food-shaping technologies (e.g., food molds and piping bags), and this high cost is a substantial barrier to the uptake [21••]. There are further concerns about the usability of the 3D food printer (e.g., time required to prepare or print foods, clean the device, reheat the food, and ensure suitable transportation of the food from production to the table to keep its shape) [21••]. Food accessibility, such as how food is arranged on a single dish, affects food choice among elderly people with dementia [44]. Food shaping need not require high cost or considerable responsibility of food provision by the provider.

## Discussion

To our knowledge, this is the first systematic review examining the association of nutrition, food shaping, and older adults with swallowing difficulties. The range of study characteristics shows the versatility of food shaping to have an impact across different populations and settings. Given there are already existing nutrition interventions in place, food shaping methods may thereby be used with other nutrition strategies to complement each other and to further optimize the nutrition of the older adult. This review enhances understanding of food shaping on nutrition, mealtime enjoyment, and participation in aged care settings, particularly showing that the combination of food enrichment and reshaping offers more varied intake which increases protein and energy intake [26] as well as enjoyment.

Above all else, the preferences of the older adult should be prioritized. Individualized food choice is important because food impacts the social and quality of life outcomes. An increasing number of aged care facilities report a lack of choice for residents on a texture-modified diet as the level of texture modification increased [38]. Effective strategies are needed to solve problems in the current food service systems in place. The role of the dietitian has been highlighted to support the best practices for preparing textured modified foods [40•]. A support system from dietary staffs, nursing managers, and cooks will help food shaping to be better accepted [41]. The staffs are already working within the constraints of food production to meet the needs of the older adults [43] and are well positioned to successfully implement food shaping.

The unique needs of the older adult should be carefully considered, noting that appearance plays a significant role in how older adults evaluate food [47]. Given that young and older adults view food shaping differently [46], further aged-care innovations should be targeted; specific age groups and the entire sensory experience should be enhanced to support food intake [43]. The mouth is the critical starting point for food breakdown for bolus preparation, transportation, digestion, and absorption of nutrients in addition to other factors such as temperature and humidity [29]. As such, the entire food journey from the placement of the food in the mouth to digestion should be assessed to support good nutrition intake.

Empowering the older adult is the key to a successful food-shaping method implementation. By providing older adults the opportunity to feed themselves, they will eat with more pleasure and increased consumption [25]. Given that the preparation of pureed foods has considerable food service challenges, commercial puree foods may be a substitute [39]. Commercial puree foods already shaped by a food shaping method should also be noted as a worthwhile option. Modified purees, in particular, have shown to take older adults longer to finish [27•] which are potentially attributed to enjoyment reflected by the increased mean oral intake. Other ambient factors have been shown to increase food intake (e.g. food color, tastiness of meal, temperature, and lighting) [24]. With factors to support the older adult’s mealtime participation in place, independence in eating may lead to empowerment and thereby a higher quality of life.

Food shaping and dietetics offer promising solutions to solve the current intricate issues associated with the provision of textured-modified food in aged care facilities. Familiarity is a critical factor in food choice and preference and can be achieved through the use of a sauce [44]. For certain cultures, older generations are used to eating dishes with sauces and this familiarity may help them accept this type of food shaping. Condiments such as sauce give care staff more options to present to the older adult with swallowing difficulty to meet nutrition needs. The use of condiments can further positively or negatively impact nutrition status depending on its content. Pouyet et al. [25] used a brown sauce for savory finger foods (vegetables, meat, and fish) for French nursing home residents. Modeling the study of Pouyet et al. [25], Kimura et al. [44] used a chocolate sauce with a finger snack of *baumkuchen* (German roll cake) for Japanese nursing home residents. In both studies, an increased consumption of food was shown with the sauce. The type of sauce, either sweet or savory, did not appear to be an enabler or barrier to intake. Although the sauces used such as chocolate may increase the amount of sugar and fat, the overall health status of the older adult should be prioritized in light of increased enjoyment and nutritional intake in the context of the prevalent issue of malnutrition in aged care settings.

The studies were conducted in several different locations globally. The benefits observed do not appear to be location dependent in terms of cultural practices, accessibility per region, or costs. It should be considered that the cultural practices of different countries were touched upon in some studies. In the study by Kimura et al., it was noted that there was no Japanese custom of eating *baumkuchen* with sauce [44]. In comparison, in the study by Pouyet et al., older generations of French people were noted to be used to eating dishes which include a sauce [25]. In both studies, an increased consumption of food with sauce was similarly shown [25, 44]. In a different study conducted in Japan, specific Japanese foods were used in the study diet such as *Chikuzenni* (simmered chicken and vegetables) [24]. This study diet was described as an efficient diet rich in nutrients in a smaller quantity of food [24]. The improved levels of mean energy and protein intake may be in relation to the smaller appearance of the food rather than the cultural practice behind it given the standard diet in comparison was also Japanese foods. In a study conducted in Denmark, traditional Danish foods were specifically noted to be used such as rye bread soup as part of the intervention [47]. Due to the diverse methods the studies used as well as the wide range of food included, additional research is needed to determine whether location was a factor in the results. Food preferences are highly influenced by a person’s culture and more information would support the future individualization of food shaping.

New food-shaping methods such as 3D food printing help to create food to meet the specific shape and dimension standards to enhance the nutrition of people with swallowing difficulty [20]. The field of 3D food printing presents several but as yet largely untested possibilities to enhance nutrition in older people with swallowing difficulties. The addition of microbeads [30, 31] and gels [32] boosted with nutrients to shaped foods is designed to increase the visual appeal of nutritious foods for individuals with swallowing difficulty. Implementing food-shaping methods such as 3D food printing may be difficult for allied health professionals to advocate due to the cost unless these devices could be shown to markedly improve nutrition and food intake along with mealtime enjoyment [21••]. It may be worthwhile in consideration of malnutrition concerns prevalent in aged care facilities. From the perspective of dietitians, fortifying 3D printed foods with vitamins, minerals, and isolated protein powders would drive the appeal and uptake of 3D printed food products [42].

The feasibility and cost-effectiveness of scaling food-shaping practices across various populations of older adults are key attributes that may determine the role of food shaping methods in aged care. However, the feasibility and cost-effectiveness are largely unexplored due to limited health economics research relating to food shaping for older people. General food-based nutrition interventions (e.g., oral nutritional supplementation, offering additional snacks, providing nutritional advice, and fortification of usual meals) in the aged care setting have shown to be clinically effective and cost-effective at improving clinical outcomes in regards to malnutrition [49]. At present, the average total spend in Australian dollars on food (including cutlery/crockery, supplements, paper goods) is estimated at $8.00 AUD per resident per day which is nearly 1.4 times less than the current food budget for prisoners and nearly three times less than the Australian average of older adults living in the community [50]. It is important to know whether food-shaping methods improve the food production process (e.g., reduce production time or food waste). More research in the health economics of food shaping is required, to fully understand the expected costs and cost-benefits to using food shaping methods in light of the current low cost of food spending allotted to the older adult in aged care.

The diversity of the studies in terms of geographic location and setting (e.g., from laboratory to aged care facilities) reveals how there are many aspects to food shaping that could make an impact on the older adult’s nutrition and health. Research from the laboratory shows how the attributes of a type of food may make a difference to the shape. Research from aged care facilities show the role shaped food may have in the real-world setting of aged care. The findings of this review emphasize the importance of both the involvement of diverse disciplines in collaboration, but also the importance of finding customizable and individual solutions for person-centered, nutritious, and enjoyable meals. Developments to enhance the efficiency of food-shaping methods are needed to enhance implementation so that older adults have access to both safe and enjoyable meals if they require texture-modified foods. With collaboration from aged care health professionals who provide food and nutrition daily as well as others interested in a positive change, food-shaping methods have potential to transform mealtimes in aged care and become a powerful tool to ensure every bite of food is nourishing and enjoyable.

### Clinical Implications for Aged Care and Nutrition in Older People

Aged care is experiencing a transformative change. In Australia, The Royal Commission for Aged Quality and Safety Nutrition recommended improvements to aged care including meeting nutrition needs and ensuring meals are desirable to eat [51]. In response, the Australian government has recently amended the *Aged Care Act 1997* (Cth) to provide high quality care which includes meeting individual nutrition needs of the older adult [52]. The infrastructure of food supply serves as an important foundation in critical times to provide food for all, including older adults. Unexpected recent circumstances such as armed conflicts may further escalate international food insecurity. Food systems and supply chains can be negatively impacted [53], and these disruptions can increase the number of malnourished people by reducing the reach of humanitarian services which support malnutrition [54]. In light of these recent events, it is notable that quality nutrition care for the older adult with swallowing difficulty is critical in obtaining good health. Given the breadth of environmental and food design factors to consider, a combination of interventions may be ideal to achieve the best outcomes.

Nutrition should be a primary focus in the pursuit of improving aged care. Food shaping can become an effective tool that brings a myriad of benefits for both the older adults and the staff. Food shaping can be incorporated in the provision of meals in various ways, from food molds to 3D food printing. The benefits in providing shaped food are potentially widespread (e.g. improved overall health, decrease need for supplements, and thus costs). In promoting food shaping as a tool to enhance nutrition of the older adult, their overall health may be improved as well as their quality of life. Food design for the purpose of good nutrition is essential for the entirety of the human lifespan, especially for the older adult who presents with swallowing difficulty.

### Strengths and Limitations of Literature Review

To increase rigor in this review, its protocol was (a) designed by all authors who are healthcare professionals across three disciplines of dietetics, speech language pathology, and nursing; experienced in working with and supporting older adults with swallowing difficulties, and (b) published prior to the comprehensive search [34], which was performed under the guidance of the university librarian. At all stages of the review, the authors consulted to discuss decisions made in interpreting the content themes of the studies. Identifying multiple types of studies for inclusion. However, this review is limited by only including studies in English since 2002. Thus, it is possible that had studies not in English and older studies been included, further insights into the impact of food shaping on nutrition, mealtime participation or quality of life might have been obtained. Given that the review is also limited by the quality of included studies, it is also important to acknowledge both strengths and limitations of studies relied in reaching the content themes in this review.

### Directions for Future Research

The novel nature of the findings of this review has illustrated the need for further research to be conducted. One overarching direction is the need for further studies to confirm positive effects in large samples [26]. More specifically, a larger sample size with randomized bolus trials and breakdown of the rheological and textural properties of foods modified [27•]. Further research with not only a larger participant group, but also a longer time period will help to ensure the validity of results [47]. Enrolling a larger number of individuals will support the collection of information to evaluate overall health status via certain parameters (e.g. pressure ulcers, infections, and changes in medications) [22].

Further research of the perspectives of residents will help determine the acceptability of shaping pureed foods [39, 46]. Perspectives of family members will be beneficial to expand the discourse on food and mealtimes in nursing homes [28]. As operational aspects are usually dependent on a few persons in the institution and are changeable in the foreseeable future, strategies should be developed to address these key persons and to spark their interest in nutrition [40•]. Focus should be placed on education of staff and how to promote flexibility safely, so that individualized needs can be met [43]. Further testing and piloting stage in long-term care setting would help determine whether food-shaping interventions such as 3D food printing is a workable, acceptable, and effective solution [42]. If further research showcases the ability to improve the nutrition and food intake along with the mealtime enjoyment, the cost of implementing food-shaping interventions such as the 3D food printer could be better justified [21••]. An improved understanding of factors contributing to the mealtime experience will be helpful in enhancing knowledge of food shaping.

Nutritious and appealing dietary solutions for older adults with swallowing difficulties warrant further research. The development of new foods for use in aged care settings is needed to enhance attractiveness of foods and improve nutrition status [25]. To develop a full picture of vegetable intake and subsequent nutrient absorption, multi-disciplinary studies are needed that combine eating behavior, bolus properties, and digestion of vegetables [45]. Other influential factors should be studied including the current state of health of the older adult, degree of swallowing difficulty, and the feasibility of the method of food shaping.

The modifications to recipes and products (e.g., the tailoring of texture-modified food production to the needs of residents) need to be supported and legitimized through improved communication and understanding among the home, corporate entities, and government regulatory bodies [41]. Research should be conducted in aged care settings assessing quality control methods to ensure appropriate textural characteristics for safe swallowing are used regularly [39]. Although aged care settings with a high number of best practices and texture-modified levels are more likely to have greater dietetic support, food shaping was reportedly the least common best practice [40•]. Therefore, there is potentially a role for dietitians to support the implementation of food shaping in meeting the older adult's nutrition needs. A mixed methods approach, considering both nutritional and mealtime enjoyment outcomes, is needed to understand all aspects of the role of food shaping for nutrition in the older adult with swallowing difficulty in the aged care setting.

## Conclusion

This review highlights that food shaping, particularly of texture-modified foods, can influence the nutrition status of the older adults including those with swallowing difficulty. The very act of eating food may be improved with shaped food, which in turn supports the individual’s nutrition intake. Food shaping, particularly into familiar shapes that look like the food content, can increase mealtime enjoyment and may address some of the physical, psychological, or physiological changes that occur with age and multiple health conditions. Aged care facilities with a higher number of best practices for texture-modified food are more likely to have greater dietetic and nutrition support, and dietitians are likely to support food shaping endeavors because they value respecting the individual’s quality of life and the right to choose in relation to texture-modified foods. Similar viewpoints in support of individual choice were seen in both older adults and their family members in emphasizing a balanced approach to combine the nutritional benefits of food with other aspects integral to the dining experience and quality of life. Food shaping has a promising future if the older adult’s individual choice is respected above all else.

## Data Availability

The data for this paper is available on request from the authors.

## Appendix. Search terms

### Web of Science

Nutrition and piping bag

Nutrition and scoop

Nutrition and plating (aged care) (dysphagia) (geriatric)

Nutrients and aged care (appearance) (food design)

Older people and improving meal times (piping bag) (food mold)

Older people and 3d food printing

Nursing home and food shaping

Nutrition and 3d food printing

Nutrient and food design and aged care

Puree and shape and nutrition

Texture and shape and nutrition

Personalized food and aged care and nutrition

Geriatric and shape and food

### PubMed

“Food design” or “food shaping”

Nutrition and scoop

Nutrition and plating and dysphagia

Nutrition and plating and geriatric

Older people and improving meal times and food mold

Older people and 3d food printing

Nursing home and food shaping

Nursing home and food design and nutrition

Nutrition and 3d food printing

Food design and nutrient and aged care

Puree and shape and nutrition

Texture and shape and nutrition

Personalized food and aged care and nutrition

Puree and shape and nutrition and older person

Geriatric and shape and food

### Cochrane Library

“Food design” or “food shaping”

Nutrition and plating and dysphagia

Nutrition and plating and geriatric

Nursing home and food design

Personalized food and aged care and nutrition

Geriatric and shape and food

### Scopus

“Food design” or “food shaping”

Nutrition and plating and geriatric

Older people and nutrients and food design

Aged care and food shaping

Geriatric and food shaping

Nursing home and food shaping

Nursing home and food design and nutrition

3d food printing and nutrition

Nutrient and food design and aged care

Puree and shape and nutrition

Texture and shape and nutrition

Personalized food and aged care and nutrition

Geriatric and shape and food

### CINAHL

“Food design” or “food shaping”

“Food design” or “food shaping” and aged or elderly or “older adult” or “older people” or geriatric

Nutrition and plating

Nutrition and aged care and appearance

Older people and improving meal times

3d food printing and nutrition

Geriatric and shape and food
